# Editorial: Updates on the pathogenesis of common variable immunodeficiency (CVID)

**DOI:** 10.3389/fimmu.2022.1130418

**Published:** 2023-01-10

**Authors:** Timi Martelius, Mikko R. J. Seppänen, Klaus Warnatz

**Affiliations:** ^1^ Inflammation Center, Infectious Diseases, HUS Helsinki University Hospital and University of Helsinki, Helsinki, Finland; ^2^ Rare Disease and Pediatric Research Centers, New Children’s Hospital, HUS Helsinki University Hospital and University of Helsinki, Helsinki, Finland; ^3^ Department of Rheumatology and Clinical Immunology, Medical Center, University of Freiburg, Freiburg, Germany

**Keywords:** inborn errors in immunity, primary immunodeficiency, primary antibody deficiencies, common variable immune deficiency (CVID), genetics

Common variable immunodeficiency (CVID) is the most common clinically manifest inborn error of immunity. Definitions of CVID have evolved over years. Infections or autoimmunity and lymphoproliferation, decreased IgG and IgA, together with impaired vaccine responses and low class-switched memory B cells have been the cardinal features of CVID.

Despite advances in genetics and discovery of numerous monogenic inborn errors of immunity under the CVID “umbrella” in the majority of CVID cases we do not understand the cause of the disease. Interestingly, from monogenic disorders we have learned that pathogenic variants of a wide variety of genes involving many different pathways can present with the clinical features of CVID. Some of the underlying genetic defects in CVID patients, like *STAT3* gain-of-function, hypomorphic *RAG* and *ADA2* demonstrate that even pathogenic variants in genes usually associated with different classes of IEIs according to the IUIS classification may clinically present as CVID.

Currently, in the remaining majority of CVID:s without a genetic diagnosis, there may be yet unknown genetic, epigenetic, or acquired causes. The defects may also affect niches outside lymphocyte populations such as the bone marrow, lymph node or thymic microenvironment.

In the absence of genetic diagnosis, alterations of differentiation, function and numbers of B and T cells, in patients with CVID may give clues on relevant pathomechanisms of the disease. For example, decreased class switched memory B-cells suggest a failure in germinal center output. This is often seen in CVIDs both without and with known genetic etiology.

This special series on the pathogenesis of CVID comprises three literature reviews on new perspectives to CVID and two original studies. First, Ho et al. summarize what is known about biomarkers in CVID and how these relate to different complications and pathogenetic mechanisms.

Fekrvand et al. review what is known about circulating B and T cell subset alterations in the reported monogenic CVID like diseases. Still, the number of patients carrying certain gene defects remains small and differentiating cellular patterns are not always found.

Gupta et al. review the data on regulatory T- and B cell subsets in CVID and their role in normal physiology. Schouwenburg et al. investigate the altered differentiation of B cells within secondary lymphoid organs of three CVID patients by combining analysis of peripheral blood B-cell subsets with histological, flow cytometric and functional analysis of B-cells in the lymph nodes and tonsils. Heterogeneity of disturbed germinal center output within CVID is well demonstrated, even by such a small patient cohort.

Last, Guevara-Hoyer et al. investigate the prevalence of CVID-associated pathogenic gene variants in non-Hodgkin lymphomas. The incidence of lymphoma in CVID is greatly increased. Pathogenic variants in the genes involved in B-cell development and differentiation have been detected in B-cell leukemias and lymphomas. Here, the authors found that 25% of non-Hodgkin lymphomas harbored variants in genes associated with monogenic CVID, *PIK3CD* and *STAT3* being most commonly involved.

Many open questions remain due to the coincidence of rarity and heterogeneity of CVID. With emerging novel technologies and increasing understanding of the human immune system, there is hope that these roads lead to new original research and better understanding of the complex pathophysiology of CVID - which until now has been is only fragmentarily understood.

## Author contributions

All authors (TM, MS, KW) jointly concepted and wrote the editorial and agree to be accountable for the content of the work. All authors contributed to the article and approved the submitted version.

